# Shade of Innovative Food Processing Techniques: Potential Inducing Factors of Lipid Oxidation

**DOI:** 10.3390/molecules28248138

**Published:** 2023-12-17

**Authors:** Aziadé Chemat, Mengna Song, Ying Li, Anne-Sylvie Fabiano-Tixier

**Affiliations:** 1 Department of Food Science and Engineering, Jinan University, Guangzhou 510632, China; 2 GREEN Extraction Team, Université d’Avignon et des Pays de Vaucluse, INRA, UMR408, F-84000 Avignon, France

**Keywords:** advantages, disadvantages, green food processing, lipid degradation, inducing factors, innovative techniques, oxidative mechanism

## Abstract

With increasing environmental awareness and consumer demand for high-quality food products, industries are strongly required for technical innovations. The use of various emerging techniques in food processing indeed brings many economic and environmental benefits compared to conventional processes. However, lipid oxidation induced by some “innovative” processes is often “an inconvenient truth”, which is scarcely mentioned in most studies but should not be ignored for the further improvement and optimization of existing processes. Lipid oxidation poses a risk to consumer health, as a result of the possible ingestion of secondary oxidation products. From this point of view, this review summarizes the advance of lipid oxidation mechanism studies and mainly discloses the shade of innovative food processing concerning lipid degradation. Sections involving a revisit of classic three-stage chain reaction, the advances of polar paradox and cut-off theories, and potential lipid oxidation factors from emerging techniques are described, which might help in developing more robust guidelines to ensure a good practice of these innovative food processing techniques in future.

## 1. Introduction

**Figure 1:**
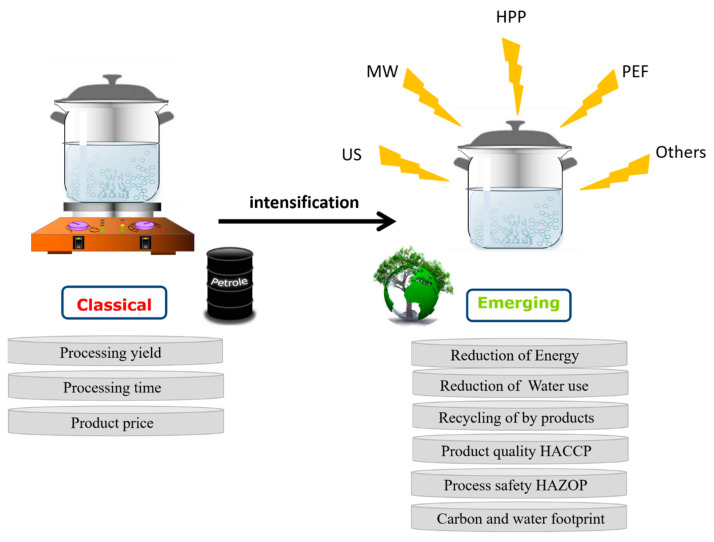
Emerging food processing technologies: evolution or revolution?

Traditional food processing technologies use heat to inactivate enzymes or spoilage microorganisms for the sake of prolonging shelf life and improving product safety and quality. However, these traditional methods produce industrial wastewater and carbon dioxide by consuming high-energy inputs of water, electricity and natural gas, resulting in low productivity and a significant environmental impact [1]. Given this, innovative technologies have continuously appeared that could cover the shortage of traditional methods to some extent like reducing cost to increase benefit, energy saving and environmental protection (Figure 1). In order to further intensify food processing with a higher quality product, it may be preferable to understand the negative impact of emerging technologies on food ingredients rather than their positive effects only [2], which would help us to make an appropriate coupling method or to enhance a current method based on its underpinning mechanism.

With the growing demand for high-quality food, the previous goal of food processing has recently been updated [3]. It is well known that food processing methods indeed have a great influence on the important attributes of foods such as texture, taste, appearance and nutrients, and future green food processing should guarantee both safety and quality [4], which requires more technological development to maximize the nutritional value and to minimize the negative effect.

Lipids are essential nutrients for human health due to their physicochemical factors such as the presence of polyunsaturated fatty acids, and they are susceptible to degradation by oxidation [5]. Lipid oxidation is a spontaneous process during food processing, which can be classified by autooxidation, photooxidation and enzymatic oxidation pathways [6]. Common initiators like heat, light and metal ions, or even a small amount of oxygen in closed storage containers, can promote lipid oxidation [7,8], some of which could be generated by innovative techniques. Despite the potential factors that might induce lipid oxidation, most studies have concentrated on the advantages of these innovative techniques instead of their side effects. For the sake of safer food of ever higher quality in future, this review focuses mainly on the potential oxidation-inducing factors of several emerging innovative techniques based on the lipid oxidation mechanism.

## 2. Mechanisms of Lipid Oxidation

### 2.1. Revisiting Initiation, Propagation and Termination

**Figure 2:**
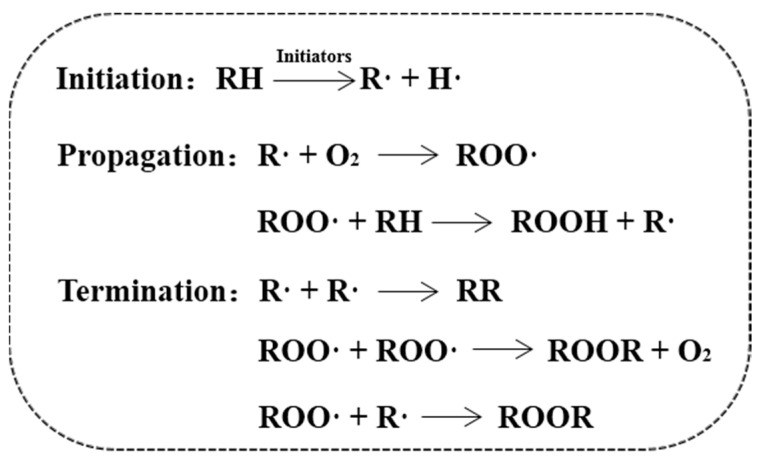
Three-stage chain reaction of lipid oxidation.

It is generally believed that oxidation begins by removing hydrogen from fatty acids or acylglycerols to form lipid radicals (LH → L∙+H∙) and undergoes a three-stage chain reaction of initiation, propagation and termination (Figure 2). However, this simplistic free radical chain reaction was found to be much more complex with the introduction of alternate chemical reactions and physical structures and properties as well. In most cases, the initiation occurs at the oil–water interface, where the interfacial properties greatly influence oxidation stability [9]. In fact, a small amount of lipid peroxides (LOOH) exists in lipids either from commercial products or extracted biological samples. Most LOOH molecules have hydrocarbon chains and hydrophilic groups, which are surface-active molecules. The decomposition of such original LOOH is assumed to provide the first radical for the oxidation initiation, especially in the presence of metal cations [10]. When these LOOH molecules reach a critical micelle concentration (CMC), the formed LOOH micelles diffuse through a continuous phase and react with transition metals near other droplets/membranes to deliver oxidation by generating LO∙ and/or LOO∙. Metal-catalyzed oxidation (LOOH + Fe^3+^ → LOO∙ + H+ + Fe^2+^) and reduction (LOOH + Fe^2+^ → LO∙ + OH^-^ + Fe^3+^) showed that metal catalysts were distributed very unevenly in the negatively charged dispersions of lipid colloids, mostly at the interface, where the surface charge could affect the lipid oxidation [11]. Similarly, thermal or UV-induced cracking (LOOH → LO∙ + OH∙) and bimolecular decomposition (LOOH + LOOH → LOO∙ + LO∙ + H_2_O) could also generate lipid-derived radicals, which can work together or compete for a reasonable starting mechanism of oxidation. With the increase in LOOH concentration in micelles, it will be more favorable for bimolecular decomposition, which can reduce the LOOH concentration to below CMC. If LOOH micelles are destroyed, oxidation can also be transferred from one colloid to another.

The propagation is usually described as a fast reaction to form peroxy radicals (L∙ + 3O_2_ → LOO∙), followed by a slow seizure of hydrogen from adjacent unsaturated lipids. Due to topological difficulties, L∙ radicals are not easily formed at the interface, where peroxy radicals (LOO∙) and alkoxy radicals (LO∙) provided from the initiation could possibly capture the hydrogen to accumulate LOOH (LOO∙ + LH → LOOH + L∙) and hydroxyl lipids (LO∙ + LH → LOH + L∙), or even lipid epoxy-hydrogen peroxide [12]. LO∙ radicals are also assumed to occur via *α*- and *β*-splitting, mainly for terminating the reaction radicals with multiple volatile secondary oxidation products. Theoretically, due to the high hydrophobicity of LOO∙, they may prevent spreading into other droplet/membrane interfaces in the aqueous phase. However, the high concentration of surfactant in the aqueous phase leads to the formation of surfactant micelles, which can be used as carriers to transfer hydrophobic substances in the aqueous phase, thus increasing the interparticle mass transfer rate of lipophilic molecules. The formation of micelles in a continuous phase of lipid dispersion may suddenly alter the mass transfer mechanism of lipid-derived oxidizing substances and thus accelerate the oxidation rate, which may explain the sudden transition from initiation to propagation [13].

### 2.2. Polar Paradox and Cut-Off Effect

**Figure 3:**
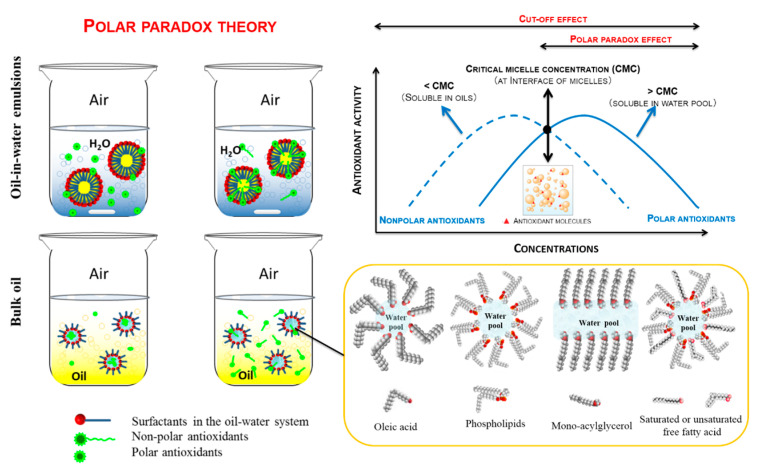
Polar paradox and cut-off effect of antioxidants in lipid oxidation.

Currently, a so-called simple, homogeneous, oil medium is actually recognized as a complex multiphase system that contains a small amount of water and various amphiphilic components, including minor compounds remaining after the refining process, and polar oxidation products as well. Polar paradox describes the phenomenon of polar antioxidants being more effective than their nonpolar homologues in bulk oils, whereas nonpolar antioxidants are more effective in water-in-oil emulsions. Therefore, the oil–water interface is considered as the oxidation site where antioxidants concentrate and take effect. Moreover, some contradictory results showed that the solubility of antioxidants in oils might have had a greater influence than the interfacial phenomenon at a lower concentration [13], which implies the limited applicability of the polar paradox (Figure 3). In oil-in-water emulsions, a nonlinear (or cut-off) effect of hydrophobic resistance to oxidation was found, i.e., the alkyl chain length of surface-active compounds strongly affected antioxidant activity, whose mechanism was logically divided into two parts: below the critical chain length and beyond the critical chain length. Below the critical chain length, it is assumed that polar antioxidants with short and medium chains are not sufficient to approach the oxidation site. When the critical chain length is reached, only antioxidants with proper hydrophobicity can be located at the water–oil interface, where antioxidants can function more effectively [14]. Beyond the critical chain length, the three following hypotheses have been proposed for this more complicated situation.

The reduced mobility hypothesis assumes that the mobility of lipophilic antioxidants decreases with an increase in alkyl chains, thereby reducing their ability to move toward oxidation sites. The binding of long-chain antioxidants to their molecular environment is stronger through hydrophobic interactions, and thus, the lower degree of freedom leads to a decrease in antioxidant activity. The increase in steric hindrance caused by the chain length makes contact between long-chain antioxidants and free radicals more difficult, which may be involved in the cut-off effect [10].

The internalization hypothesis describes that increasing a hydrocarbon chain from a medium chain to a long chain keeps antioxidants away from its interface. With an increase in emulsifier content, the cut-off effect of medium-chain esters (e.g., butyl, octyl and dodecyl esters) in water-in-oil emulsions gradually disappeared while long-chain esters entered the aqueous phase from an oily core, which was more effective in preventing lipid oxidation at the interface [15].

The tendency of forming long-chain aggregates is greater than that of moving toward the boundary mask, thus reducing the antioxidant concentration at the oxidation site. The self-aggregation hypothesis proposes that amphiphilic antioxidants with long alkyl chains and polar heads can self-assemble into micelles, layered structures and other associated colloids to form stable monolayers at the air–water interface. Among the low-concentration emulsifiers (lower than CMC), the only physical and chemical process possible for the accumulation of lipid-soluble antioxidant compounds in the aqueous phase is the self-polymerization of antioxidants through micellization [14].

Similarly, these hypotheses might also be applicable to bulk oils, where CMC would be the significant factor to regulating and controlling the degree of micellization. Since the effectiveness of polar antioxidants is better in oil systems, this may inspire a novel strategy for lipid antioxidation through reverse micelle-assisted extraction, which enables the coexistence of both lipid- and water-soluble antioxidants in the same lipid system. Although the assistance of most innovative techniques could definitely improve this efficiency, its side effects on reverse micelle formation like the generation of free radicals or initiators, and temperature, also deserve attention for optimal micellization in lipids, resulting in the best antioxidative effect.

## 3. Description of Lipid Oxidation Induced by Emerging Processing Techniques

As previously described, one common and important factor in lipid oxidation is active free radical formation. Table 1 presents the general characteristics of some emerging food processing techniques. Although the pros and cons of these emerging techniques were included in most studies, it was hard to find lipid oxidation descriptions for all technical studies. According to the mechanism of lipid oxidation, innovative food processes involving potential inducing factors are summarized as follows.

**Figure 4:**
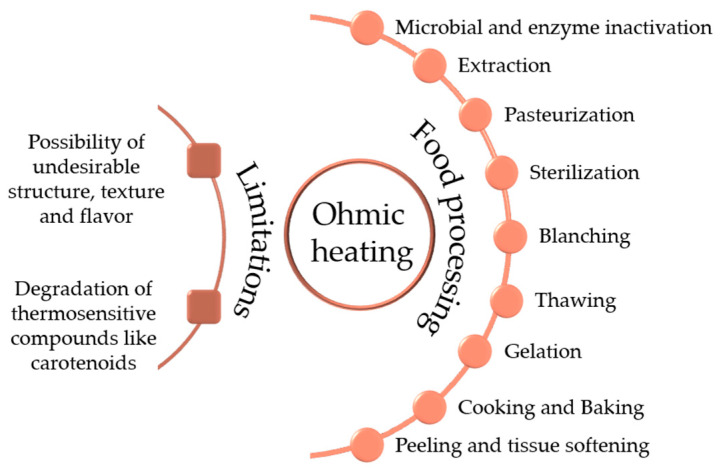
Applications of ohmic heating with its impacts on food components.

As Figure 4 illustrates, the applicable scope of ohmic heating in food processing has been widely expanded upon. Compared to conventional methods, ohmic heating can maintain the quality (e.g., main antioxidants, color, acidity, etc.) of fruit and vegetable juices better, especially for the condition of being under vacuum [16,17,18,19]. Nevertheless, electrochemical reactions resulting from ohmic heating application may lead to the generation of free radicals and undesirable texture. Furthermore, concerning the different electric behaviors of various antioxidants, a fundamental understanding of the impact of a moderate electric field on them still needs to be explored to obtain the desired electric effects.

An atmospheric plasma device can form active ions through molecules in ionized gas, which might initiate oxidation reactions. Among them, ozone is a three-atom oxygen molecule formed by the interaction between diatomic oxygen molecules and oxygen radicals, and the formation of O–O free radicals requires the plasma system to provide energy [20]. At the same time, ozone is a very active and unstable substance, which can easily decompose into hydroxyl radicals, hydrogen peroxy radicals and superoxide radicals that have high oxidation properties [2].

High pressure generally does not initiate lipid oxidation, and free radicals formed by cleavage are not affected by increased pressure. However, pressure can affect the formation of covalent bonds during the propagation stage [2]. Cheftel and Culiolib [21] observed that myoglobin and oxymyoglobin were transformed into Fe^3+^ forms at higher than 350 MPa to catalyze lipid oxidation. Bolumar et al. [22] found that high pressure could damage the cell membrane and release intracellular free radicals or their precursors, which actually promote lipid oxidation.

Using a pulse electric field (PEF) has shown its different effects on lipid oxidation in oi-rich products depending on the operating conditions [2]. However, many chemically active compounds can be produced by discharging or by reacting a food matrix with electrodes. Liquids close to the electrode surface produce an electrolytic effect and eventually produce active chemicals such as hydrogen peroxide, hydroxyl radical or chloride ion. In addition, under the effect of PEF action, oil will produce alkyl radical, alkoxy radical, superoxide anion radical and so on, among which superoxide anion radical is very active in being transformed into other active hydroxyl radicals, hydrogen peroxide radicals and so on.

Lipid oxidation induced by γ radiation is a typical free radical reaction. The gamma ray is a kind of high-energy ray containing a large amount of ionizing radiation energy, which induces the H loss reaction of methylene connected with double bonds in unsaturated fatty acids and forms free radicals [23]. Radiation can degrade water molecules, leading to the generation of oxidation (e.g., hydroxyl radicals) and reduction products (e.g., hydrogen atoms) [24]. Among them, hydroxyl radicals can easily convert myoglobin into ferromyoglobin and even convert iron in heme into its free state, forcing it to become the major catalyst for lipid oxidation [25].

**Table 1:** Characteristics of emerging food processing techniques.

Technology	Principle	Processing Mechanism	Advantages	Limitations	Reference
Radiation	A photon of no mass, capable of penetrating material	Forms positively and negatively charged ions by interacting with food molecules; these unstable particles rapidly convert into highly active free radicals and react with food ingredients	✓ Improves processing efficiency ✓ Reduces enzyme activity ✓ Strong penetration ✓ Avoids secondary pollution ✓ High reliability ✓ Suitable for mass production	✧ High cost ✧ Radiation risk ✧ Insufficient consumer awareness of radiation ✧ Loss of nutrition ✧ Causes changes in oxidative flavor ✧ Difficult to detect	[26]
Plasma	An ionized gas consisting of particles produced by free radicals, ions, electrons and other discharges; available at atmospheric or sub-atmospheric pressures by discharge or strong ultraviolet radiation	When oxygen is present as part of a gas, reactive oxygen species in the plasma may cause food quality to decline	✓ Low water consumption ✓ Low operating temperature ✓ Low cost ✓ Inactivated pathogens ✓ Enzyme inactivation ✓ Changed hydrophilicity/hydrophobicity	✧ Promotes oxidation of certain food ingredients ✧ Reduces food quality ✧ Shortens shelf life	[27]
High pressure	A food preservation technology that puts food into a sealed, high-strength pressure vessel, exerts pressure at a certain temperature and maintains it for a period of time, also known as ultrahigh pressure, or high hydrostatic pressure	To delay or accelerate the rate at which a particular reaction occurs, together with changes in physical properties and effects on equilibrium processes	✓ High retention rate of functional active components ✓ Increases mass transfer rate of liquid oil ✓ Increases permeability of solvent in cells ✓ Increases diffusion of secondary metabolites	✧ Small effect on food enzyme activity ✧ Presence of some microorganisms ✧ Expensive equipment investment	[28]
Pulsed electric field	Very short pulses of high-voltage direct electric current generated between two electrodes, leading to electroporation and non-thermal modification of the tissue structure	Cell destruction in a food matrix without damaging food properties, which can improve mass transfer and cause electroporation and inactivation of the microbial cell wall	✓ Increases mass transfer efficiency and extraction yield ✓ Shortens the processing time ✓ Very little heating of the food corresponding to less effect on the color, nutrient content and flavor of food ✓ Reduces the loss of thermally sensitive compounds ✓ Time- and energy-saving	✧ No effect on enzymes and spores ✧ Difficult to use with conductive materials ✧ Only for liquids ✧ Electrolysis may adversely affect food ✧ High cost of investment and low equipment capacity	[29]
Ohmic heating	Conduction and convection heat is generated internally within the food mass due to tissue’s electrical resistance.	Motion of charged particles on the conductive food materials between electrodes through the passage of electric current; heating takes place throughout the entire volume of the food	✓ Rapid and uniform heating ✓ No need for large heating surfaces ✓ Suitable for particulate–liquid mixtures ✓ Possible to have near-instantaneous startup and shutdown of the heating unit	✧ Foods used for processing should be pumpable ✧ Foods should have a good electrical conductivity ✧ The process variables should be selected cautiously	[16]
Instant controlled pressure drop	A high-temperature and high-pressure treatment, the raw material is treated by saturated steam in a short time and then suddenly pressure drops to vacuum	Changes in structural characteristics (porosity, surface area), increased diffusivity and permeability within plants and availability of certain active molecules	✓ Reduction in processing time ✓ Instantaneous reduction in temperature ✓ Prevention of further thermal degradation ✓ High quality of extracts	✧ High energy consumption	[30]
Compressed liquefied gas	When a gas is liquefied, its physical and chemical properties become better	Using low-pressure liquefied gas as solvent, changing the process selectivity by adjusting the pressure at mild temperature, thus changing the solvent extraction efficiency	✓ Low pressure ✓ Substitutability ✓ Improves enzyme catalytic ability ✓ Reduces solvent use ✓ Time- and cost-saving	✧ Solubility of polar compounds ✧ Frequent maintenance	[31]
Supercritical fluids	Changes in physical properties through pressure and/or temperature adjustment beyond critical values	The density of supercritical fluid is close to that of liquid, resulting in its dissolving power being close to liquid, viscosity, close to gas, and diffusivity, between liquid and gas	✓ Increases mass transfer ✓ Improves selectivity ✓ Reduces the use of organic solvents ✓ Solvent-free residue of extracts	✧ Miscibility with polar compounds ✧ Professional requirement ✧ High startup investment	[4]
Ultrasonication	Non-thermal technique using frequencies in the range of 20–100 kHz at power levels of 10–1000 W/cm^2^	The collapse of cavitation bubbles generates very high localized pressure (100 MPa), temperature (5000 K) and forces sufficient to destroy cell walls	✓ Improves heat transfer ✓ Inactivates microorganisms in liquid foods	✧ Free radical formation ✧ Off-flavor ✧ Metallic taste ✧ Structural modification	[32]

## 4. Invisible Effects of Emerging Techniques on Lipid Degradation

### 4.1. Ultrasound

#### 4.1.1. Principle

Ultrasound has found numerous applications in the food industry, such as processing, extraction, emulsification, preservation, homogenization, etc. [33]. Ultrasound (US) refers to mechanical waves which have the property of spreading in elastic media such as liquids [34]. The ultrasonic wave is mainly characterized by four physical parameters, namely the frequency (Hertz), ultrasonic power (W), wavelength (cm) and ultrasonic intensity (W·cm^−2^). It is worth mentioning that ultrasonic intensity (UI) is directly related to ultrasonic power (UI = P/S; P: power (W) and S: the emitting surface (cm^2^)).

US frequencies range between 20 kHz and 10 MHz, above the human hearing range (from 16 Hz to 20 kHz). High frequencies (from 2 MHz to 10 MHz) and low ultrasonic power (P < 1 W) are applied in the case of diagnostic US essentially used for therapeutic purposes such as medical imaging. In this power range, there is no destructive effect into the medium. The desired effect is only to characterize the medium by measuring the submitted modification of the ultrasonic wave during its propagation into the medium [32]. Power US is characterized by low frequencies (from 20 kHz to 100 kHz) and high ultrasonic power (P > 10 W). Contrarily to diagnostic US, high power promotes physical and chemical effects by creating sufficient interaction between the ultrasonic wave and the elastic medium. This frequency range is widely valorized in several fields such as food processing and extraction of natural products. Physical impacts are essentially observed at low frequencies (from 20 kHz to 100 kHz), while different chemical impacts can be observed in the extended range of power US frequencies (up to 2 MHz), mainly in the formation of radicals [35].

US-induced impacts can be attributed to the cavitation phenomenon referring to bubble formation, growth and implosion during its propagation into an elastic medium [34,36]. The benefit of the cavitation phenomenon is related to the concentration of acoustic energy in small volumes (bubbles) and its conversion in extreme physical conditions of temperature and pressure. While passing through an elastic medium, a spatial and temporal variation in acoustic pressure is induced into the medium, where an oscillatory movement can therefore be observed on the surface.

Undergoing a succession of compression and rarefaction phases, the medium’s constitutive molecules can be displaced from their equilibrium position. During the compression phase (negative acoustic pressure), intermolecular distance is significantly reduced leading to possible collision with the surrounding molecules. During the rarefaction phase (positive acoustic pressure), intermolecular distance increases dramatically [37]. Thus, voids are created between the constitutive molecules once their cohesive forces are exceeded by a higher ultrasonic power. These voids, also called bubbles, are formed from vapors or gases initially present in the elastic medium. Vapors and/or gases entering bubbles are partially expelled during the compression phase, resulting in a final increase in bubble size after many cycles of rarefaction/compression phases. In other words, the bubble volume increases with each cycle until it reaches a critical size. At this stage, bubbles collapse during the compression cycle [34,35,37,38]. Bubble implosion results in the creation of hot spots with extreme conditions of temperature (up to 5000 K) and pressure (up to 5000 atm), which explains their extremely high physical and chemical reactivity [34,36,37,38].

#### 4.1.2. Effects on Food Lipids

**Figure 5:**
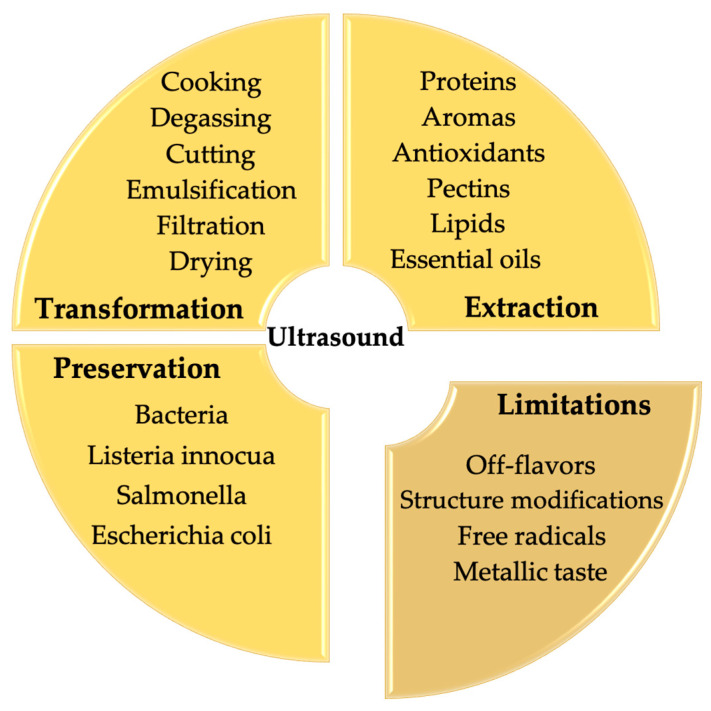
The potential restrictions and/or chemical effects generated by cavitation phenomena.

Although ultrasound is able to produce beneficial modifications in food quality parameters (e.g., viscosity and homogenization), the physicochemical effects of ultrasound treatment might also result in quality impairments of food products by the appearance of off-flavors, modifications in physical parameters and degradation of major and minor compounds. Due to these critical temperature and pressure conditions, allied to the formation of radicals during sonocavitation, some alterations in food components have been reported during ultrasonic treatment. Acoustic cavitation can produce radicals in a liquid medium and molecules such as OH and H radicals can accumulate at the surface of the cavitation bubble, which can be responsible for initiating the formation of degradation products that can also trigger radical chain reactions and provoke substantial quality defects in those products [39]. The potential restrictions and/or uses of the chemical effects generated by cavitation phenomena are shown in Figure 5.

An increasing number of reports in the literature concern modifications in high-lipid-containing food products. Table 2 summarizes the effects of ultrasound on high-fat food products, as well as the experimental conditions used in those studies. Lipid deterioration is of great economic importance in the production of lipid-containing food products. Oxidation of unsaturated lipids not only produces unpleasant odors and flavors but can also decrease the nutritional quality and safety by the formation of secondary reaction products in foods. In food products, lipid autoxidation is often referred to as rancidity, which describes the off-flavors obtained by subjective organoleptic evaluation of the product [40]. Lipid oxidation can also destroy essential fatty acids and produce oxidized polymers and toxic compounds [41]. The lipid oxidation phenomenon depends on several complex reaction mechanisms, which are related to the lipid’s structure and the medium conditions under which the lipids are present. Some determining variables to lipids’ oxidative stability are the number and nature of the present unsaturation, the type of interface between the lipids and oxygen, exposure to light and heat, and the presence of pro- or antioxidants.

### 4.2. Microwaves

#### 4.2.1. Principle

Nowadays, microwaves have not only gained in popularity for defrosting, heating or cooking, but are also used in food processing such as drying, thawing, tempering, cooking, baking, sterilization, blanching and extraction. Microwave radiation has many advantages; this process is completed in a few seconds or minutes with high reproducibility, reducing the extraction time and energy normally needed for conventional heating.

Microwaves are electromagnetic waves with a frequency range from 0.3 GHz to 300 GHz, i.e., they span the range of wavelengths from 1 m to 1 cm. Their waves are between radio frequencies and infrared radiation on the electromagnetic spectrum. Industrial applications in food processing have grown steadily since the frequencies of 2.45 GHz and 915 MHz have become more common. Microwaves are composed of an electric and magnetic field and thus represent electromagnetic energy. This energy is a type of innocuous radiation that creates the molecular motion of ions by the rotation of dipoles but has no effect on molecular structure. This dipole rotation comes from alternative movement of polar molecules which try to line up with the electric field. Many collisions due to the agitation of molecules generate energy release, which results in rapid heating. Thus, microwave radiation comes from dissipation of the electromagnetic waves in the irradiated material. The dissipated power in the medium depends on the dielectric properties and the electric field strength.

The mechanisms of microwaves and conventional heating are different. Microwave heating transforms electromagnetic energy into thermal energy, which starts from a heat source and transfers to a medium by conduction, convection or radiation in conventional heating. This phenomenon can be explained by the Fourier heat equation, where *ρ*, C_p_, κ, T and t represent the specific density (kg·m^−3^), specific heat capacity (J·kg^−1^·K^−1^), thermal conductivity (W·m^−1^·K^−1^), temperature (K) and time (sec), respectively.

(1) ρCp∂T∂t+κ∇2T+PV=0

#### 4.2.2. Effects on Food Lipids

In contrast with conventional heating, microwaves allow a rapid rise in temperature, a volumetric heating, and the maximum temperature of the irradiated material depends only on the rate of heat loss and power applied. The distribution of the electric field is not homogeneous in the irradiated material and “hot spots” appear if heat production is faster than heat transfer. At this moment, for highly viscous media such as oils, the degradation induces oxidative processes to vegetable oils, leading to quality and nutritional losses, as well as lower bioactive properties and physical changes [60]. Table 2 summarizes the effects of microwave heating on the degradation of vegetable oils and the experimental conditions used in those studies. Cerretani et al. [57] investigated the effect of microwave radiation on the formation of reactive free radicals that rapidly reacted with atmospheric oxygen to produce secondary oxidation products. The formation of secondary oxidation products in olive oil was determined by testing for the p-anisidine value, which showed a significant increase after 3 min of microwave heating. The peroxide value as another oxidative index was also evaluated, which greatly decreased after up to 6 min of heating. These preliminary results show that microwave energy may induce oxidation in olive oil.

Moreover, Borges et al. [59] studied the effects of microwave heating on the composition and physicochemical properties of baru and soybean crude oils. They concluded that both oils became oxidized after 3 min of heating with a 94% decrease in tocopherol content, corresponding to a reduced antioxidant activity by half, and the oxidative stability was reduced by about 72%, accompanied by the loss of its typical yellow coloration. In the same way, Karrar et al. [64] investigated the impact of microwave heating on the lipid composition and the oxidative stability of gurum seed oil, whose results showed that triacylglycerol and diacylglycerol decreased with microwave heating (800 W) after 2, 4 and 6 min, respectively, compared with the untreated sample. This same trend was observed for the change in tocopherol content, which has several benefits to overall human health. Another study aimed at evaluating the physicochemical properties and oxidation stability of castor oil using microwave-assisted solvent extraction (MAE) from castor seed [105]. The oil from the MAE was more viscous and had a higher acidic value compared to that of the Soxhlet extraction as the reference. The increase in acidic value may be attributed to the hydrolysis of triacylglycerols by microwaves which produce more free fatty acids.

### 4.3. Ohmic Heating

#### 4.3.1. Principle

Ohmic heating is also known as electric resistance heating, which is a technique based on the passage of alternative current (50–100 Hz) through food material in order to generate internal heat (i.e., Joule effect). Parameters such as the voltage and the frequency of electric current and electrical conductivity can affect the characteristics of food components since they determine the heating rate. The first industrial application of ohmic heating began in 1920 with milk pasteurization in a continuous process. This technique is particularly fit for viscous products, liquid foods and the concentration process especially for fruits with a high electrical conductivity value which leads to heating in a few seconds. Many studies revealed that ohmic heating is superior to conventional heating in terms of energy and time saving [106]. In addition, the use of low frequencies between 50 and 60 Hz increases electrochemical reactions and the erosion of electrodes, where the contact between the electrodes and the material food is a critical aspect of the process.

The advantages of ohmic heating include uniform and volumetric heating, reduced processing time and thermal damage to thermolabile components like vitamins, bioactive ingredients and color parameters [107], as well as non-contact between the food material and hot surfaces. Ohmic heating allows for the conversion of electrical energy into thermal energy, which can be used as an intermittent batch process or in a continuous flow system [108,109]. Several studies showed that ohmic heating had little effect on the oxidative degradation of vitamin C [110], whose degradation depends on the treatment time, the type of electrode and the voltage gradient.

#### 4.3.2. Effects on Food Lipids

Ohmic heating is unsuitable for foods with low electrical conductivity such as those with a high fat content. Hence, studies concerning the impact of ohmic heating on fatty acid profiles are still scarce. Al-Hilphy et al. [111] reported an ohmic-based oil extraction from fish waste, which showed better quality under appropriate processing conditions than the conventional method. Fresh Gac aril is very susceptible to oxidation and degradation; Aamir and Jittanit [112] studied the effect of ohmic heating on Gac aril oil extraction in comparison with conventional heating. The experiments were conducted using three extraction stages at 50 °C with the selected ratio of Gac aril powder to the solvent and time for each stage. With the ohmic method, the extraction efficiency and the content of carotenoids in the Gac aril oil were enhanced with the porous and ruptured microstructure of oil-extracted raw material. Kumari et al. [113] optimized their process parameters (900 V/m, 85 °C for 10 min) to maximize the recovery of sesame oil. Although the ohmic heating treatment of sesame slightly increased the FFA in the oil, all FFA values were below the maximum permissible limit for all treatment combinations. In addition, Kuriya et al. [114] investigated the effect of ohmic heating on the quality of blueberry-flavored dairy desserts, where different electric field strengths (1.82, 3.64, 5.45, 7.30 and 9.1 V/cm) at 60 Hz were used and compared to a conventional heat treatment (90 °C/3 min) as the control. The type of processing and the electrical field had no significant impact on the fatty acid profile.

### 4.4. Plasma

#### 4.4.1. Principle

Plasma is often referred to as the fourth state of matter. It is an ionized gas composed of free electrons, ions, reactive atoms, neutral fractions and photons that are in a metastable state with a net charge of approximately zero. According to the temperature of electrons, plasma can be divided into low-temperature and high-temperature plasma [115]. More specifically, low-temperature plasma can be divided into thermal plasma and non-thermal or cold plasma according to its thermodynamic equilibrium [116]. Moreover, cold plasma exhibits thermodynamic imbalance at two temperatures, i.e., ions and neutral molecules remain at low temperatures (slightly higher than room temperature), while the temperature of electron gas is about 10^4^ K [117]. Therefore, the cold plasma system used in food processing is kept at a relatively low temperature, which is very beneficial to the food processing industry.

Dielectric barrier discharge (DBD) and plasma jets are commonly used in food processing. The DBD device consists of two metal electrodes while at least one electrode is covered by a dielectric barrier, which acts as a stabilizing material to avoid any arc transitions and create a large amount of microdischarge for uniform processing. The plasma jet device consists of two concentric electrodes while the inner electrode is usually connected to power at a high frequency, resulting in the ionization of working gas, which presents as a “jet-like” nozzle [118].

#### 4.4.2. Effects on Food Lipids

Plasma is an emerging food processing technology, among which non-thermal plasma, especially atmospheric plasma, has received widespread attention in the food industry [119]. It is an accelerated oxidation technology with great potential to predict lipid oxidation phenomena and/or oxidation stability. This plasma can standardize the control of lipid-accelerated oxidation in complex food matrices with the production of high-concentration active substances such as singlet oxygen, hydroxyl radicals, atomic oxygen, ozone and excited molecular nitrogen [78]. Unfortunately, these active substances, and free radicals in particular, can also initiate lipid oxidation by hydrogen abstraction from lipid molecules.

Gas in the electric field can accelerate the movement of charged ions and free electrons. These accelerated particle collisions with other molecules lead to energy sharing, displacement reactions and charge exchange, resulting in several free radicals. When discharging to feed gases containing N_2_ and O_2_ molecules, their collision with electrons leads to a series of reactions, forming N_x_O_y_, O_3_ and peroxy dimer. The collision of electrons (e^−^) with O_2_ leads to the formation of solitary oxygen atoms in the discharge zone (e^−^ + O_2_ → 2O + e^−^), which are then attacked by reaction O_2_ to produce ozone (O + O_2_ + M → O_3_ + M), where M is O, O_2_ or O_3_. However, ozone and singlet oxygen will promote potential lipid oxidation in foods. When water is present in the feed gas, it causes OH, H_2_O_2_ and H formation, which in turn may inhibit O_3_ formation [71].

Table 2 summarizes some effects of plasma on lipid oxidation in foods, as it is known that the type and content of a lipid are closely related to its oxidation. Gavahian et al. (2018) [71] found that the thiobarbituric acid reactive substance (TBARS) value of brown rice after 20 min of atmospheric plasma treatment was higher than that of white rice, indicating that plasma is more suitable for foods with a relatively low fat content. Bahrami et al. [72] showed that treating wheat flour with plasma for 1 or 2 min significantly reduced the content of free fatty acids and phospholipids in the wheat flour, and plasma-treated wheat flour decreased the content of linoleic acid by 100% compared to untreated wheat flour. Thirumdas et al. [120] observed that the peroxide content in peanuts and walnuts treated with 60 kV plasma increased by 20% in their production of oxidative rancidity. Lee et al. [73] found that dielectric barrier discharge for 10 min did not cause the oxidation effect in packed chicken breast. The oxidation stability of the chicken breast was however better than that of red meat plasma, which might have been related to the higher fat content in the meat. Choi et al. [74] found that corona spray discharge caused lipid oxidation, resulting in an increase in the TBARS value during storage while it could improve the sanitary quality of semi-dry squid. The high unsaturated fatty acid content in squid is sensitive to lipid oxidation [121], which may be related to primary oxidation products and active substances produced by further plasma reactions. The free fatty acids and other primary oxidation products generated from the drying process make the lipids in squid more susceptible to oxidation by plasma.

Moreover, lipid oxidation is associated with input power, processing time and storage. The TBARS value of bresaola (i.e., dried and aged bacon) samples treated by one-minute atmospheric pressure plasma with air-conditioned packaging (30% of O_2_ and 70% of Ar) increased from 0.15 to 0.35 mg/kg [75]. Atmospheric pressure plasma at 100 W for 1.5 min had a negative effect on lipid, though it could improve the microbial safety of bacon [76]. The TBARS value of bacon increased after 7 days of storage, indicating that the presence of oxygen in the carrier gas accelerated the lipid oxidation rate. Plasma treatment did not affect the lipid oxidation level in canned ham due to the presence of nitrite and ascorbic acid [119]. Yong et al. [77] reported that the TBARS value of cheddar cheese could be influenced by plasma treatment time, where lipid oxidation could be reduced by optimizing process parameters. Plasma treatment could accelerate lipid oxidation, especially for the formation of volatile secondary oxidation products like aldehydes and ketones. The concentration of volatile secondary oxidation products in plasma-treated olive oil samples increased significantly [78]. Similarly, the secondary oxidation products in plasma-treated fish oil significantly increased compared with untreated fish oil [79].

### 4.5. High Pressure

#### 4.5.1. Principle

Pressure is a basic thermodynamic variable corresponding to temperature. Thermal effects during a high-pressure process (HPP) can cause changes in material volume and energy [122]. Combined net effects during an HPP may be synergistic, antagonistic or superimposed. Reactions such as phase transitions or molecular redirection depend on temperature and pressure, which cannot be treated alone. The previously mentioned HPP principles as follows [123].

- Isostatic principle: Regardless of the geometry and size of the food, the pressure is assumed to be uniform and equal in all directions of the food composition.
- Le Chatelier’s principle: Any phenomena (phase transition, changes in molecular configuration, chemical reactions) accompanied by a decrease in volume are enhanced by pressure, which will facilitate a system’s transition to the lowest volume.
- Microscopic ordering principle: An increase in pressure at a constant temperature enhances the order of a given material molecule. Therefore, pressure and temperature antagonize molecular structures and chemical reactions.
- Arrhenius relationship: As with heat treatment, various reaction rates in the HPP process are also affected by the thermal effect during pressure treatment. Net pressure–heat effects can be synergistic, superimposed or antagonistic.

#### 4.5.2. Effects on Food Lipids

The most pressure-sensitive biological components are lipid systems [124]. Indeed, the melting temperature of triglycerides can increase by more than 10 °C per 100 MPa, and thus lipids in a liquid state at room temperature crystallize under high-pressure treatment [12]. Bolumar et al. [22] found that free radical formation would not occur at pressures below 400 MPa, which can be considered as a threshold in HPP treatment. The kinetics of free radical formation followed a zero-order reaction at pressures below 600 MPa, whereas that at higher treatment pressures was more aligned with a first-order reaction with a reaction rate of 0.016–0.07 μM/min [125].

Pressure affects not only the physical properties of food components (e.g., surface tension, density, viscosity and thermal properties, etc.) and dynamic equilibrium processes, but also the rate of lipid oxidation by slowing down or accelerating the reaction. Hebishy et al. [84] observed a higher oxidation rate for emulsions treated by an ultrahigh pressure of 200 MPa compared to those treated by 100 MPa, especially for those containing 1% or 2% of whey protein isolate, which may have been due to the decreased ability of whey protein to protect oil droplets. With the increasing pressure in the ultrahigh pressure treatment, the temperature at the outlet of the homogeneous valve increased, resulting in the over-processing phenomenon. Whey proteins were partially denatured or aggregated, leading to large polymeric dissociation, which could allow more proteins to gather on the droplet surface and prevent oxidation better [85]. Pereda et al. [87] found that the content of malondialdehyde and hexanal was much lower in milk under 300 MPa compared to that of 200 MPa. Wang et al. [86] also found that the TBARS values of treated fat samples at 400 MPa and 600 MPa were much higher than those at 200 MPa, indicating that lipid oxidation increased with pressure.

Although the temperature generated by high-pressure processing is considered low, it is sufficient enough to affect various nutrients and bioactive molecules [2]. The emulsification of multiple oils (i.e., sunflower, camel and fish oils) by microfluidization at the pressure of 21–138 MPa using sodium caseate as an emulsifier could lead to an increase in oxidation stability [87]. The increased temperature of water-in-oil emulsion during pressure treatment could lead to the binding of lipids to proteins during storage, resulting in a reduction of oxidation products. Bolumar et al. [22] found thresholds for the formation of free radicals at 25 °C and 400 MPa and 5 °C and 500 MPa, respectively. Above these thresholds, free radical formation increased with the increasing pressure, temperature and time. It is believed that the synergistic effects of high pressure and temperature could promote lipid oxidation.

In addition, there are many factors affecting oxidation, such as the oil content, physical structure of emulsion (e.g., size and specific surface area of droplets), emulsifier and emulsion type, etc. [83,126]. As Table 2 summarizes, Fuentes et al. [88] reported the oxidative stability difference in two dry-cured ham types under a high-pressure treatment of 600 MPa, namely in the flank (lower fat content) and hip (higher fat content), indicating that unsaturated lipids in the flanking samples were more easily oxidized corresponding to their high TBARS value. Atares et al. [80] used a high-pressure jet homogenizer of 30 MPa to determine the structure and oxidative stability of water-in-oil emulsions prepared with sunflower oil in the presence of the flavonoids rutin and whey protein as emulsifiers. The droplet size decreased after high-pressure homogenization, whereas the emulsion structure stability increased, thus reducing lipid oxidation. Nakaya et al. [127] found that the oxidation stability of lipids in an emulsion could be enhanced by reducing the droplet size. Phoon et al. [81] used high-pressure homogenization (0.1~137.9 MPa) to form a water-in-oil emulsion (4%, *w/v*), which showed a poor oxidation stability due to its larger droplet size when exposed to oxygen directly. Ultrahigh-pressure homogenization is a novel antioxidant technique for the production of fine, stable submicron emulsions [128]. Soybean oil and conjugated linoleic acid emulsions (20%, *v/v*) containing soy protein isolate (4%, *w/v*) as an emulsifier were studied [82], indicating that the emulsion treated by ultrahigh-pressure homogenization (100~300 MPa) had the smallest particle size with the best oxidation stability. Furthermore, lipid oxidation decreased with increasing oil content under constant pressure according to the change in the TBARS value, which is consistent with previous findings [129]. This may have been due to the fact that the water-soluble pro-oxidant components decreased proportionally with the increased oil phase in the emulsion, thus reducing the number of free radicals and slowing down lipid oxidation [130]. Compared to other oil content, emulsions with 10% of oil content treated at an ultrahigh pressure also had poor physical stability, which might have been due to their link with oxidation stability [131]. The mechanism of HPP-induced cholesterol oxidation remains unclear. The most supported hypothesis is related to cell membrane damage, which can induce free radical formation through the synergistic action of denatured proteins [132]. Furthermore, applying very high pressure (>800 MPa) can also form free radicals and promote lipid peroxidation, resulting in cholesterol oxidation [125]. It is believed that the increase in the oxidation rate may be due to the increase in the interface area, which leads to the increase in contact between the oil and peroxide.

### 4.6. Pulse Electric Field

#### 4.6.1. Principle

PEF technology applies a high voltage pulse in a specific and short amount of time, resulting in a high electric field with electroporation phenomena occurring in the treated material placed between two electrodes [133]. A transmembrane potential difference is formed on the cell membrane under the action of an applied electric field. When the electric field strength of the transmembrane exceeds the threshold, the voltage shrinkage force causes a local dielectric breakdown of the membrane, resulting in a pore as a conductive channel [134]. Due to high electric field pulses, the cell membrane increases membrane permeability by expanding existing pores or generating new ones, which may be permanent or temporary depending on the operating conditions [29]. The mechanism of electroporation is mainly based on the voltage contraction force that affects the cell membrane. Hence, the pulsed electric field technique is considered as a pretreatment process for the disintegration of vegetative organisms [135], which illustrates the electrical, reversible and irreversible breakdown of the cell membrane.

#### 4.6.2. Effects on Food Lipids

PEF is a non-thermal food preservation technology mainly used in liquids. Compared to traditional hot barrel sterilization, PEF can inactivate most pathogenic or spoilage microorganisms, which has the advantages of maintaining food freshness effectively, having an impact on enzymatic activity and is energy-saving. Minimizing the loss of taste, color, texture, nutrition and thermal-sensitive functional components in food has attracted increasing attention in recent years [9,136,137,138]. PEF is among the emerging technologies that have been successfully applied in various low-viscosity liquid foods such as milk, soy milk, pea soup, egg liquid and juice beverages [89]. However, few studies concerning the effects of PEF on food composition have been reported, especially in food lipids [139]. Therefore, understanding the role of PEF technology in electrochemical reactions and lipid oxidation is necessary for further development of the food processing industry [95].

PEF treatment can change the permeability of cells, which makes meat components such as lipids easier to oxidize or to promote the reaction between enzymes and their substrates. It can also change fatty acids and volatile components and ultimately affects the shelf life of food [93]. Moreover, Pataro et al. [140] showed that metal ions released from pulsed electric fields led to electrode contamination or corrosion, and even lipid oxidation at the end. Table 2 summarizes some effects of PEF on lipid oxidation in foods. Zeng et al. [89] observed that the acidic value of PEF-treated peanut oil after storage at 40 °C for 100 days was lower than that of untreated peanut oil, while the carbonyl value during this storage period decreased with the increase in the electric field intensity, indicating that PEF treatment could inhibit the rate of lipid oxidation. Arroyo et al. [90] found that the malondialdehyde content of PEF-treated fresh frozen chicken breast increased but there was no significant difference in the TBARS value for different conditions. Cortes et al. [91] also noted that the peroxidase of PEF-treated samples was partially inactivated while the TBARS value was not significantly changed.

Furthermore, Ma et al. [92] found that PEF-treated lamb meat would not produce lipid oxidation immediately. However, the malondialdehyde content in the treated sample after 7 days of storage was higher than that in the control, though the product quality was still acceptable (<2 mg malondialdehyde/kg sample). Notwithstanding, Faridnia et al. [93] found that the lipid oxidation of PEF-treated beef muscles was significantly enhanced, where the TBARS value was higher than that of the non-PEF treated samples. PEF treatment made thawed-from-frozen meat more prone to lipid autoxidation caused by the release of metal ions in iron complexes. The thawed-from-frozen samples accumulated the most malondialdehyde content after a storage period of 18 days. High-voltage PEF-treated boned beef samples exhibited a higher lipid oxidation rate compared to those treated with low-voltage PEF at the end of the storage period [3], which is probably because of the high thermal energy generated during the high-voltage PEF treatment that could reduce the antioxidant capacity of meat and accelerate the lipid oxidation rate during storage. Moreover, no significant effect was found on the acidic value of PEF-treated oleic acid and lecithin samples after storage [95,96]. Nevertheless, the change in both the peroxide value and carbonyl value at different degrees was influenced by the electric field intensity and storage time, indicating that PEF treatment did induce the oxidation of oleic acid and lecithin.

### 4.7. Radiation

#### 4.7.1. Principle

The effects of radiation can be divided into direct and indirect effects. The direct effect is due to the nonspecific collision of radiation photons with atoms in microbial molecules. Radiation disintegrates key biomolecules such as DNA, RNA, enzymes and membrane proteins [141]. It also induces the formation of DNA photoproducts, namely cyclobutane pyrimidine dimer and pyrimidine (6–4) pyrimidone photoproducts, which inhibit transcription and replication and inactivate microorganisms [142,143]. The indirect effect is due to the effect of free radicals produced during irradiation hydrolysis. Ionizing radiation can generate sufficiently high energy to activate chemical reactions in many food systems. Radiation first ionizes one electron in the effluent, producing highly active substances such as hydroxyl radicals and hydrogen peroxide, and then forms many intermediates which can react with each other or with other components in the system. Many intermediates produced during this time have high chemical activity [144]. Therefore, the indirect effect of irradiation on microbial inactivation depends on the water availability in food [141].

#### 4.7.2. Effects on Food Lipids

Electromagnetic waves (e.g., visible, x, γ, ultraviolet, infrared, etc.) and electrons can be used in food processing with the advantages of having uniform heating, high heat transfer efficiency, less mass loss, being energy-saving, and having a prolonged shelf life and improved safety. It has been reported that the shorter the wavelength, the better the thermal penetration effect [98]. The free radicals formed by irradiation have an important effect on the oxidative stability of foods with high fat content, but generally, they have no effect on the nutritional value of foods. Similar to the results of lipid changes observed using conventional methods, irradiation accelerates oxidative decay in foods [145]. Food products with either a higher lipid or unsaturated fatty acid content are more prone to oxidation reaction, which is mainly caused by free radicals formed in the indirect action of radiation [146]. The higher the irradiation dose, the higher the excitation level, and thus more free radicals are produced to easily enhance lipid oxidation and color change [147].

The lipid oxidation rate increases with the radiation dose. As Table 2 presents, an oxidation rate ranging from 0 to 50 kGy increased faster than that of 50–100 kGy [96]. A significant positive correlation between the radiation dose and peroxide value was found with a correlation coefficient of 0.908 [97]. The peroxide value of peanut oil extracted from infrared radiation-treated seeds was significantly higher than that from original seeds [98], which may be due to the temperature increase during the roasting process. According to Lee et al.’s study [148], the radiation dose of 5.0 kGy greatly increased the oxidation of soybean oil, cottonseed oil, corn oil and linoleic acid. The concentration of both primary and secondary oxidation products increased with the increase in γ radiation dose [99]. Both primary and secondary oxidation products accumulated in peanut oil under a γ radiation of 8 kGy, where the content of secondary oxidation products increased faster [100]. Cashew nuts (*Anacardium occidentale* L.) radiated at higher doses (7 kGy) could be oxidized to form aldehydes and ketones as well [102]. The content of these volatile secondary oxidation products was also found to increase significantly in peanut and pistachio oils using the same radiation dose [103].

The oxidation stability index is affected by many factors, such as fatty acid composition and antioxidant content. Total antioxidant capacity increases as the roasting temperature increases. Hence, the storage stability of peanut oil from an infrared radiation pre-baking treatment significantly improved compared to the control [98]. Similarly, some Maillard reaction products generated from heating treatment can also improve the antioxidant capacity of oil [149]. However, γ radiation shortens the induction period of crude peanut oil and reduces the oxidative stability, though the total tocopherol content is positively correlated with the induction period [99].

Radiation can also cause a content change in endogenous antioxidants in oils to some extent, like tocopherols and phenolic compounds. The polyphenolic content of peanut oil extracted from infrared radiation roasted seeds increased by 62.20% whereas the contents of total tocopherol and three tocopherol congeners decreased significantly compared to oils from raw peanuts [98]. The degradation of tocopherol exceeded the oxidative protection of Maillard reaction products when the temperature increased from 147 °C to 157 °C. The decrease in γ-tocopherol content was affected differently by the instantaneous γ radiation of 5.0 kGy [99]. The loss of α-tocopherol in soybean oil was as high as 92.3% with γ radiation of 3.0 KGy [103]. Irradiation could significantly decrease the tocopherol content, among which α- and δ-tocopherol degraded the most while γ-tocopherol resistance to degradation was the best [104]. 

## 5. Conclusions and Perspectives

The content of active compounds and the absence of denatured molecules are generally two main factors used to determine the quality of food lipids. During the processing of food containing considerable level of lipids, lipid degradation may occur depending on the process conditions like high temperature, long-term treatment, presence of light, oxygen, metal ions and free radicals. Although the aforementioned innovative food processing methods in accordance with the green extraction concept aim to obtain non-denatured and biodegradable end products without contaminants with added values, their accompanying negative effects require additional attention. The key to further investigating such techniques in both academia and food industries is to select or combine together the appropriate technique for the future good manufacture practice. Furthermore, some alternative hypotheses on lipid oxidation reactions and mechanisms still require evidence, which is of paramount importance for the optimization of processing conditions for the sake of high-quality products with maximum economic value and minimum lipid oxidation. Furthermore, reliable and robust equipment of good applicability is also necessary to guarantee the reproducibility of products and stable quality control.

## Figures and Tables

**Table 2:** Effects of emerging techniques on lipid oxidation.

Food Matrix	Experimental Conditions	Observations	References
**Ultrasound**			
Virgin olive oil	20 **A**, 400 **B**, Titanium alloy microprobe **C**, 5 **D**, Spectrophotometer **F**, Rancimat method **G**	Ultrasound probe, irradiation time, duty cycle and pulse amplitude are the most influential variables on the acceleration of the olive oil oxidation process	[42]
Refined sunflower oil	20 **A**, 150 **B**, Titanium alloy probe **C**, 0.5–2 **D**, 20 **E**, UV spectroscopy, GC and GC/MS **F**, Sonication **G**	Increase in peroxide value, decrease in polar compounds and appearance of off-flavors	[43]
Sunflower oil	20/47 **A**, 450 **B**, Titanium alloy probe **C**, 60 **E**, 20/60 **F**, UV spectroscopy, GC and GC/MS **F**, Emulsification and sonication **G**	Sonodegradation identified with off-flavor compounds	[44]
Soybean germ and seaweed oils	19/25/40/300 **A**, 80 **B**, Titanium cup horn, immersion horn and cavitating tube **C**, 30/60 **D**, 45 **E**, GC/FID **F**, Extraction **G**	Slight oxidation with decrease in the relative percentage of unsaturated fatty acids, irrespective of the degree of unsaturation	[45]
Kiwi seed oil	80 **B**, Titanium horn **C**, 30 **D**, 50 **E**, GC/MS and sensory evaluation **F**, Extraction **G**	Partial lipid degradation found with the presence of off-flavors	[46]
Bleached olive oil	20 **A**, 750 **B**, Immersible probe **C**, 13–43 **D**, 30–70 **E**, HPLC and SPME/GC/MS **F**, Bleaching **G**	Increase in peroxide value and acid value, losses in α-tocopherols and minor changes in fatty acid composition; the oil flavor partly deteriorated after long treatment	[47]
Soybean oil	20 **A**, 90–180 **B**, Probe **C**, 0.5–3 **D**, 25 **E**, GC, SEM **F**, Extraction **G**	Increase in saturated fatty acids, decrease in unsaturated fatty acids and the oxidation percentage was 3.4%	[48]
Flaxseed oil	20 **A**, 600 **B**, Microprobe **C**, 5/10/20 **D**, GC/MS, GC **F**, Extraction **G**	Minor effect on fatty acid losses, peroxide levels increased and free radicals may have also been generated; organic solvent may have limited oxidation	[49]
Palm and sunflower oils	66 **A**, Ultrasonic ring transducer cell **C**, 15 **D**, 45 **E**, Microscope and GC/MS **F**, Crystallization **G**	Appearance of benzene as one of the oxidation products in a very small quantity	[50]
Chocolate mousse	25 **A**, 150 **B**, Bath **C**, 2 **D**, 25 **E**, Color, Sensory analysis **F**, Food preparation **G**	Darker color of sonicated samples, decrease in viscosity and apparition of off-flavors	[51]
Sunflower oil	40 **A**, - **B**, Titanium sonotrode **C**, 3 **D**, Ion chromatography and sensory test **F**, Cutting **G**	A short ultrasonic treatment was sufficient to generate a remarkable off-flavor	[52]
Kolkhoung (*Pistacia khinjuk*) kernel oil	24 **A**, 100 **B**, Titanium sonotrode **C**, 30/40/50 **E**, GC and HPLC **F**, Extraction **G**	The fatty acid and oxidation of the oil were not affected by the ultrasound but the temperature	[53]
Castor oil	20 **A**, 130 **B**, Standard probe **C**, 35 **D**, 70 **E**, CLSM and SEM **F**, Soxhlet extraction and thermosonication extraction **G**	More stable to oxidize during thermosonication due to low iodine and peroxide values	[54]
Rapeseed	40 **A**, 73.5/105 **B**, Bath **C**, 30 **D**, 37–49 **E**, GC/FID, pressure DSC and TEM **F**, Pretreatment before oil pressing **G**	Unfavourable changes were observed in the oxidative stability of the oil after seed sonication	[55]
Microalgae (*Heterochlorella luteoviridis*)	20 **A**, 72 **B**, Probe **C**, 10 **D**, 30 **E**, GC/MS, GC/FID and TEM **F**, Extraction **G**.	No oxidation process was observed; carotenoids acts as an antioxidant in preserving polyunsaturated fatty acids	[56]
**Microwaves**			
Olive oils	2.45 **A**, 720 **B**, 1.5–15 **D**, 145–313 **E**, HPLC **F**, Heating **G**, Domestic microwave oven **H**	Microwave heating induced oxidative alterations, especially in extra virgin olive oil and olive oil	[57]
Refined peanut, high-oleic sunflower and canola oils	2.45 **A**, 720 **B**, 1.5–15 **D**, GC/FID and DSC **F**, Heating **G**, Domestic microwave oven **H**	Different degrees of lipid thermooxidation induced by microwaves in vegetable oils were observed	[58]
Baru and soybean crude oils	1000 **B**, 1–15 **D**, GC-FID and color **F**, Heating **G**, Domestic microwave oven **H**	Increase in peroxide value, color change	[59]
Soybean germ and seaweed oils	100 **B**, 30/60 **D**, 60/120 **E**, GC/MS **F**, Extraction **G**, Open and closed vessel or under pressure **H**	Higher yields were achieved with closed-vessel irradiation at 120 °C with negligible lipid oxidation, as well as combined ultrasound/microwave irradiation	[45]
Olive oil	1000 **B**, 1–10 **D**, 30 **E**, GC-FID and HPLC **F**, Cooking **G**, Domestic microwave oven **H**	Addition of vegetable extracts to improve the stability of olive oil	[60]
Extra virgin olive oil	700 **B**, 15 **D**, 50–225 **E**, Raman spectroscopy and GC/FID **F**, Cooking **G**, Microwave oven **H**	A progressive degradation of carotenoids in extra virgin olive oil was observed at 180 °C	[61]
Sunflower and corn oils	700 **B**, 2–10 **D**, 80–158 **E**, GC-MS and HPLC **F**, Extraction **G**, Microwave oven **H**	Increase in primary and secondary oxidation products, fatty acid content and tocopherol content	[62]
Virgin olive, refined sunflower and peanut oils	1100 **B**, 15 **D**, - **E**, Spectrophotometer, GC-FID **F**, Cooking **G**, Microwave oven **H**	Increase in the trans isomers of unsaturated fatty acids	[63]
Gurum seed oil	800 **B**, 2–6 **D**, 74–146 **E**, Spectrophotometer, color and GC-MS **F**, Extraction **G**, Microwave oven **H**	Oxidative stability increased with microwave heating for different times	[64]
Mashhadi melon, Iranian watermelon, pumpkin and yellow apple seed oils	1000 **B**, 1–15 **D**, GC/FID and oil quality analysis **F**, Home heating and cooking **G**, Microwave oven **H**	Oil quality decreased with longer exposure to microwave heating, resulting in the formation of primary and secondary oxidation products	[65]
Black cumin seed oil	180/540/900 **B**, 1.5/3/4.5 **D**, 25 **E**, Rancimat device, Spectrophotometer, SEM **F**, Pretreatment before extraction **G**, Microwave oven **H**	Inverse relation between the microwave power and the time of the oxidative stability; microwave radiation degraded susceptible bioactive compounds	[66]
Flaxseed oil	2.45 **A**, 180/360/540 **B**, 5/10 **D**, Color, Spectrophotometer and Rancimat **F**, Roasting **G**, Microwave system **H**	Formation of Maillard reaction products during roasting led to a change in oil color	[67]
Chia seed oil	2.45×10^6^ **A**, 180–900 B, 15 **D**, GC and HPLC, Spectrophotometer **F**, Roasting **G**, Industrial microwave device **H**	Microwave roasting could cause significant changes in the physicochemical properties of chia oil like losses in its bioactive components	[68]
Poppy seed oil	2.45×10^6^ **A**, 720 **B**, 25 **D**, GC/FID and Spectrophotometer **F**, Roasting **G**, Microwave oven **H**	Microwave roasting cast negative effects on the nutritional and functional attributes of the seed and oil	[69]
Soybean oil	10–60 **D**, 150–250 **E**, NMR, Pressurized DSC and viscometer **F**, Irradiation **G**, Sophisticated microwave oven **H**	Microwave irradiation increased the oil viscosity due to the formation of a cyclic ring structure with polymerization	[70]
**Cold plasma**			
White and brown rice	250 **B**; 20 **D**	TBARS increased after 20 min of treatment	[71]
Wheat flour	15/20 **B**; 1/2 **D**	The content of free fatty acids and phospholipids decreased significantly	[72]
Chicken breast	DBD, Peak power: 100, average power: 2 **B**; <10 **D**	No lipid oxidation observed	[73]
Semi-dry squid	Pulsed corona discharge; 20 **I**; 1.5 A; 10 **D**	TBARS value increased	[74]
Bresaola	15.5/62 **B**; 5/0.33 **D**	TBARS value increased	[75]
Bacon	14000 **A**, 75/100/125 **B**; 1.5 **D**	Higher TBARS values after 7 days of storage	[76]
Cheddar cheese	DBD, Peak power: 100, average power: 2 **B**; 10 **D**	TBARS value increased	[77]
Olive oil	DBD; 6 **I**; 60 **D**	The concentration of secondary oxidation products increased	[78]
Fish oil	DBD; 6 **I**; 60 **D**	The concentration of oxidation products increased significantly	[79]
**High pressure**			
Sunflower oil	30 **J**	Oxidation reduction.	[80]
4% (*w/v*) water-in-oil emulsion	0.1~137.9 **J**	The oxidation stability of the crude emulsion was poor	[81]
Soybean oil and conjugated linoleic acid (20%, *v/v*) emulsion	15 **J**; 15 **J**, High-temperature short-time conditions; 200 **J**	The oxidation stability followed the order of ultrahigh-pressure homogenization > conventional homogenization > conventional homogenization + high-temperature short-time conditions	[82]
10–20% (*w/v*) water-in-oil emulsion	15 **J**; heat, 15 **J**; 100–300 **J**	20% of the water-in-oil emulsion had the best oxidation stability	[83]
10–50% olive oil	100, 200 **J**, 25 **E**; 5000 rpm, 20 **E**; 15 **J**, 60 **E**	100 J of high-pressure homogenization stability	[84]
15% sunflower seed oil + 5% olive oil	100, 200 **J**, 15 **J**	The treated emulsion had high oxidation stability, and the 100 J treatment especially was the best	[85]
Yak body fat	100–600 **J**; 4, 15 **E**; 20 days	Samples treated under lower pressure had good sensory acceptability; high-pressure treatment had a catalytic effect on lipid oxidation	[86]
Milk	200, 300 **J**	High concentration of secondary oxidation products for the 300 J treated group	[87]
Fresh meat	400–800 **J**; 5–40 **E**; 0–60 **D**	High pressure, temperature and time synergistic effects promoted an increase in free radicals	[22]
Dry-cured ham	600 **J**; 2 **E**; 120 days	Samples with high muscle fat content were unstable	[88]
**Pulse electric field**			
Peanut oil	20/30/40/50 **I**; 1 **A**; 40 μs	Lipid oxidation inhibition	[89]
Chicken breast	0.01/0.055/0.11 **A**; 7.5/10/12.5 **I** (fresh samples) 14/20/25 **I** (frozen samples); 20 μs	No lipid oxidation observed	[90]
Vegetable beverage	20–35 **K**; 100–475 μs	No lipid oxidation observed	[91]
Cooked lamb meat	1–1.4 **K**; 20 μs; 0.09 **A**	No lipid oxidation observed	[92]
Beef muscles	1.4 **K**; 20 μs; 0.05 **A**	Lipid oxidation observed	[93]
Cold boned beef	Low-voltage PEF (2.5 **I**, 0.2 **A**, 20 μs); high voltage PEF (10 **I**, 0.2 **A**, 20 μs)	Higher degree of lipid oxidation in high-voltage pulsed electric field samples	[3]
Oleic acid	25–35 **K**; 400 μs	Lipid oxidation observed	[94]
Lecithin	0–35 **K**; 0–800 μs	Lipid oxidation observed	[95]
**Radiation**			
Flaxseed and Tung oils	0/50/100 **L**	Oxidation acceleration	[96]
Rapeseed oil	2/4/7/10 **L**	No secondary oxidation product, which was positively correlated with the peroxide value	[97]
Peanut oil	Infrared shortwave radiation; 150 **E**; 25/40/55/70 **D**	Improved oxidation stability of the extracted oil	[98]
Peanut oil	2.5/5/7.5/10 **L**; 6 months at room temperature	Induction period and tocopherol content were negatively correlated with irradiation dose; radiation and storage increased the production of oxidized compounds	[99]
Peanut	4/6/8 **L**	Irradiation was an effective tool for peanut oil preservation.	[100]
Raw unpeeled almond kernels	1/1.5/3/5/7 **L**	Volatile off-flavor compounds increased with the increase in irradiation dose.	[101]
Cashew	1/1.5/3/5/7 **L**	Volatile compounds such as aldehydes, ketones and alcohols increased, corresponding to lipid oxidation	[102]
Vegetable oil	1/2/3 **L**; additional tocopherol	The antioxidant activity decreased significantly	[103]
Red meat	0–9.4 **L**	The content of tocopherol decreased significantly	[104]

**A**: Frequency (kHz), **B**: Power (W), **C**: Type of ultrasonication, **D**: Exposure time (min), **E**: Temperature (°C), **F**: Main analytical method, **G**: Type of treatment, **H**: Type of microwave apparatus, **I**: Kilovolt (KV), **J**: Megapascal (MPa), **K**: Kilovolt/centimeter (KV/cm), **L**: Kilogray (kGy). GC/MS: Gas chromatography mass spectrometry; GC: Gas chromatograph; GC-FID: Gas chromatography-flame ionization detection; HPLC: High-performance liquid chromatography; SPME: Solid-phase micro-extraction; CLSM: Confocal laser scanning microscopy; FESEM: Field-emission scanning electron microscopy; DSC: Differential scanning calorimetry; TEM: Transmission electron microscopy; NMR: Nuclear magnetic resonance; DBD: Dielectric barrier discharge; TBARS: Thiobarbituric acid reactive substance.
